# Pesticidal Activity of Citrus Fruits for the Development of Sustainable Fruit-Processing Waste Management and Agricultural Production

**DOI:** 10.3390/plants14050754

**Published:** 2025-03-01

**Authors:** Hisashi Kato-Noguchi, Midori Kato

**Affiliations:** Department of Applied Biological Science, Faculty of Agriculture, Kagawa University, Miki, Kagawa 761-0795, Japan

**Keywords:** agronomic utilization, allelopathy, citrus fruit waste, essential oil, food processing, limonene, pesticide, waste management

## Abstract

The annual global production of citrus fruits is over 150 million tons, and 40–50% of the citrus fruits are processed into juices and other products. The processing generates a large amount of waste and causes environmental issues. In order to reduce the environmental impacts, several approaches for the waste management of citrus fruits were proposed. The citrus fruit waste contains several functional compounds, but the extraction of these functional compounds requires adequate production facilities. The waste is not suitable to carry for long distances due to the high percent of water content and its heavy weight, and it is not suitable to store for a long time due to the occurrence of fermentation. Some of the approaches target the use of waste in the proximity of the processing factories. The application of citrus fruit waste for crop production in the agricultural fields close to the faculties is one of the possible management options. The evidence of citrus fruit waste as herbicidal, nematocidal, insecticidal, and anti-fungal materials has been accumulated in the literature over three decades. Several compounds involved in these functions have also been identified in the citrus fruits. However, there has been no review article focusing on the pesticidal activity of citrus fruits against weeds, herbivore insects, parasitic nematodes, and pathogenic fungi. This is the first review article providing an overview of such activities and compounds involved in the functions of citrus fruits.

## 1. Introduction

The *Citrus* genus, belonging to the Rutaceae family, is an evergreen shrub thought to be native to Southeast Asia. Taxonomy of the *Citrus* species is a challenging scientific problem, dividing into three taxa to 156 taxa [1,2,3]. However, many of the species are considered to be derived from natural and/or artificial hybridization among a small number of true species [4,5,6].

*Citrus* is one of the most extensively cultivated fruit species in the world, with 4000 years of cultivation history, and is typically grown on either side of a belt around the 35° N and 35° S latitudes, such as China, USA, Mexico, India, Brazil, and Spain [5,7,8]. Annual global production of citrus fruits is increasing, with 30 million tons in 1960 and 158 million tons in 2020 [5,8]. Although many kinds of citrus fruits are available in the global markets, *Citrus sinensis* (L.) Osbeck (orange) is the most cultivated species among *Citrus* species, and the annual global production of the *Citrus sinensis* fruits was 75 million tons in 2020 [8], which is 47% of total citrus fruit production in 2020. The annual global production of other major citrus fruits in 2020 was 38.6 million tons for *Citrus reticulata* Blanco (mandarin); 21.3 million tons for *Citrus limon* (L.) Osbeck (lemon), including *Citrus aurantiifolia* (Christm.) Swingle (lime); and 9.3 million tons for *Citrus maxima* (Burm.) Merr. (pomelo) including *Citrus paradisi* L. Grapefruit Group (grapefruit) [8].

About 50–60% of the total citrus fruits are consumed as fresh, and the remaining 40–50% of the citrus fruits are subjected to industrial processing to make various commodities, such as juices, jams, canned products, flavoring agents, and cosmetics, with various level of facilities, depending on the location and factory [5,8,9,10]. The processing generates a large amount of waste, which is about 55–70% of the total fresh fruit weight, including peel (7–40% of total fresh fruit weight and 55–75% of total dry fruit waste weight), pulp (20–55% and 22–33%), and seeds (0–5% and 0–9%), depending on the *Citrus* species [9,11]. On a dry weight basis, therefore, the peel is the most abundant, followed by the pulp and seeds. In addition, all harvested fruits do not satisfy the quality requirements for the fresh fruit markets and industrial processing, and these low-quality fruits are discarded as waste [9,12]. Such a large amount of citrus fruit waste causes environmental issues [8,9,10,11].

Citrus fruit waste is acidic (pH 3–4) and contains a high percentage of water and organic matter [13,14]. The traditional strategy of citrus waste management is mainly incineration and landfilling, which are large expenses [14]. High levels of nitrogen and carbon may be released into the environment through incineration and landfilling. The traditional strategy of citrus waste management does not meet environmental concerns and is no longer suitable for citrus waste management [12,14].

In order to reduce environmental impacts, several alternative means were proposed for citrus waste management. Citrus waste contains several functional compounds, such as pectin, carotenoids, polyphenols, dietary fiber, vitamins, proteins, and organic acids [15]. However, these compounds must be extracted from citrus waste and manufactured into functional foods, food additives, medicines, cosmetic products, etc. [16,17,18,19,20,21]. Bioethanol and biogas are able to be produced from fruit waste due to their high concentration of carbohydrates [14,22,23,24,25,26]. These processes of citrus fruit waste for the extraction of the functional compounds and the generation of bioethanol and biogas require adequate production facilities and transportation of the waste to the production facilities [11,14,21,27,28,29,30]. Adequate facilities are not always available in local citrus fruit processing industries. The waste is not suitable to carry for long distances due to the high percent of water content and its heavy weight, and it is not suitable to store for a long time due to the occurrence of fermentation [9,26,31]. Citrus fruit waste can be used as animal feed due to its high nutrient values [32,33]. Fresh citrus waste is available only for the citrus processing season and must be carried from the processing facility to the animal farm. Therefore, the dried citrus waste is more suitable for the animal feed [10,34,35,36]. However, the drying process of citrus waste requires facilities that are not always available, especially in developing countries [10,37,38].

The application of citrus waste as organic fertilizer is also one of the possible waste management options [39,40,41,42]. As citrus waste is not cost-effective for transportation to distant sites, the main target of the waste as an organic fertilizer is for agricultural production in the proximity of the processing factories [40,43]. The citrus waste compost, which was made from the fresh *Citrus sinensis* fruit waste, increased the growth of tomato (*Solanum lycopersicum* L.) and zucchini (*Cucurbita pepo* Linnaeus) [42]. The *Citrus sinensis* fruit waste also increased the wheat (*Triticum durum* Desf.) production [44]. The fresh *Citrus sinensis* fruit waste was incorporated into *Citrus sinensis* orchards with and without calcium hydroxide, which is for the neutralization of low pH waste, and the fruit yield, soil microbiota, and soil physicochemical characteristics were monitored [43]. A mixture of the calcium hydroxide eliminated soil acidification. The soil pH in the plots without adding the calcium hydroxide was low during the first three months after the waste incorporation. However, the pH became neutral after 8 months of the incorporation. The waste application increased the soil microbial activity and the soil chemical potential, such as the soil aeration, cation exchange capacity, and water retaining capacity for both plots, irrespective of calcium hydroxide. The fruit yield also increased significantly with the fruit waste application [43].

It was reported that the peel powder and the extracts of citrus fruits, such as *Citrus sinensis* and *Citrus junos*, suppressed the growth of several weed species [45,46]. The essential oil obtained from the fruit peel of *Citrus sinensis*, *Citrus aurantium,* and *Citrus reticulata* suppressed the germination and growth of some weed species [47]. These observations suggest that the citrus fruits may be allelopathic and contain certain allelochemicals. The evidence of allelopathy in citrus fruits, including the essential oil, has been accumulated over three decades. Allelopathy is the chemical interaction between donor plants and receiver plants through certain compounds defied as allelochemicals. Allelochemicals inhibit the germination and growth of receiver plants. Donor plants produce and accumulate allelochemicals in some plant parts until the release of allelochemicals into the nearby receiver plants [48,49,50,51,52]. Therefore, the allelochemicals accumulated in the plants are potentially extractable, and the extracts can be applied for foliar sprays as herbicides. The plant parts can also be applied as soil-addictive materials, and the allelochemicals are released during the decomposition process of the plants into the soil and act as herbicides [53,54,55]. Therefore, the citrus fruit processing waste, including the essential oil, is potentially useful for weed management purposes. In addition, the citrus fruits were reported to show the nematocidal, insecticidal, and anti-fungal pathogen activity [56,57,58]. Weeds, parasitic nematodes, herbivore insects, and pathogenic fungi are significant restrictions on crop production [59,60]. Therefore, citrus fruit waste may be applied to control weeds, parasitic nematodes, herbivore insects, and pathogenic fungi in agricultural production. However, there has been no review article focusing on the allelopathic, nematocidal, insecticidal, and anti-fungal pathogen activity of citrus fruits. The literature was searched using a combination of the predominant online search engines, Scopus, ScienceDirect, and Google Scholar, and all possible combinations of citrus waste with the following words: allelopathy, allelochemical, nematocidal activity, insecticidal activity, anti-pathogen activity, phytotoxic, management, and organic farming. As a result, the citrus fruit peel and its essential oil were most investigated, and the *Citrus* species *Citrus sinensis*, *Citrus reticulata*, *Citrus limon*, *Citrus aurantiifolia*, *Citrus maxima*, *Citrus paradisi, Citrus margarita* (Lour.) Swingle (kumquat), and *Citrus junos* (Makino) Siebold ex Tanaka (yuzu) were reported to have allelopathic, nematocidal, insecticidal, and/or anti-fungal pathogen activity. It is the first review article providing an overview of such activities and compounds involved in the activities of these *Citrus* species.

## 2. *Citrus sinensis* (Orange)

### 2.1. Herbicidal Activity of Citrus sinensis Fruits

*Citrus sinensis,* containing sweet orange, Valencia orange, blood orange, and navel orange, is thought to be a hybrid between *Citrus reticulata* and *Citrus maxima* and to be distributed from Southern China [61,62]. The fruits of *Citrus sinensis* are widely processed into juices and other foods, including functional foods. Orange juice is the most consumed citrus juice in the world [9,11,12]. The processing generates a large amount of waste, which is about 55% of the total fresh fruit weight, including peel (65% of total dry fruit weight), pulp (32%), and seeds (2%) [9].

Weed infection causes significant crop yield loss, and adequate weed management is necessary to achieve the potential yield in agricultural production [63,64]. When the seeds of *Phaseolus vulgaris* L. and two weed species, *Sonchus oleraceus* L. and *Digtaria ciliaris* (Retz.) Koel, were sown into the soil after the peel powder of the *Citrus sinensis* fruits was applied onto the soil surface, the growth of the weed species was suppressed, while the growth and yield of *Phaseolus vulgaris* were increased [46].

Aqueous methanol extracts of the *Citrus sinensis* fruit peel inhibited the germination and growth of *Amaranthus hybridus* L. and *Pennisetum glaucum* (L.) R.Br with a concentration-dependent manner [65]. The aqueous ethanol extracts of the *Citrus sinensis* peel suppressed the growth and cell division of *Allium cepa* L. roots [66]. Aqueous extracts of the *Citrus sinensis* whole fruit waste inhibited the germination and growth of *Chenopodium album* L. under the Petri dish and pot conditions. *Lactuca sativa* L. (8 cm in height) was transplanted to the field soil, and spontaneous weed emergence on the field soil was monitored for 70 days. During the monitoring period, the aqueous extracts of the *Citrus sinensis* fruit waste were applied onto the soil surface every two weeks (a total of three times). The emergence of four forb species, such as *Euphorbia serpens* Kunth, *Cirsium arvense* (L.) Scop., *Portulaca oleacea* L., and *Abutilon theophrasti* Medik., and three grass species, such as *Setaria italica* P. Beauv., *Echinochloa crus-galli* (L.) P.Beauv., and *Sorghum halepense* (L.) Pers., were observed in the control pots, while a few weeds were observed in the treatment plots. In addition, an increase in the fresh and dry weight of *Lactuca sativa* was observed in the treatment plots. Limonene was the main constituent in the extracts, and its concentration was 1542 mg/L [67].

These observations suggest that the application of the powder and the extracts of *Citrus sinensis* fruits and its fruit waste suppressed the germination and growth of several weed species, indicating that *Citrus sinensis* fruits are allelopathic and contain certain allelochemicals, including limonene. These allelochemicals are extractable with methanol, ethanol, and water and can suppress plant germination and growth [68,69,70,71,72,73,74,75]. Therefore, the allelochemicals in the powder and extracts of the *Citrus sinensis* fruits and fruit waste may inhibit the emergence and growth of the weed species. However, *Lactuca sativa* was not inhibited in the field observation of Ugolini et al. [67]. One of the reasons may be that *Lactuca sativa* had already germinated and established and was able to obtain a relatively large quantity of resources, such as nutrients, water, and light, because of lower competition with a few weeds compared to the control treatments.

**Figure 1:**
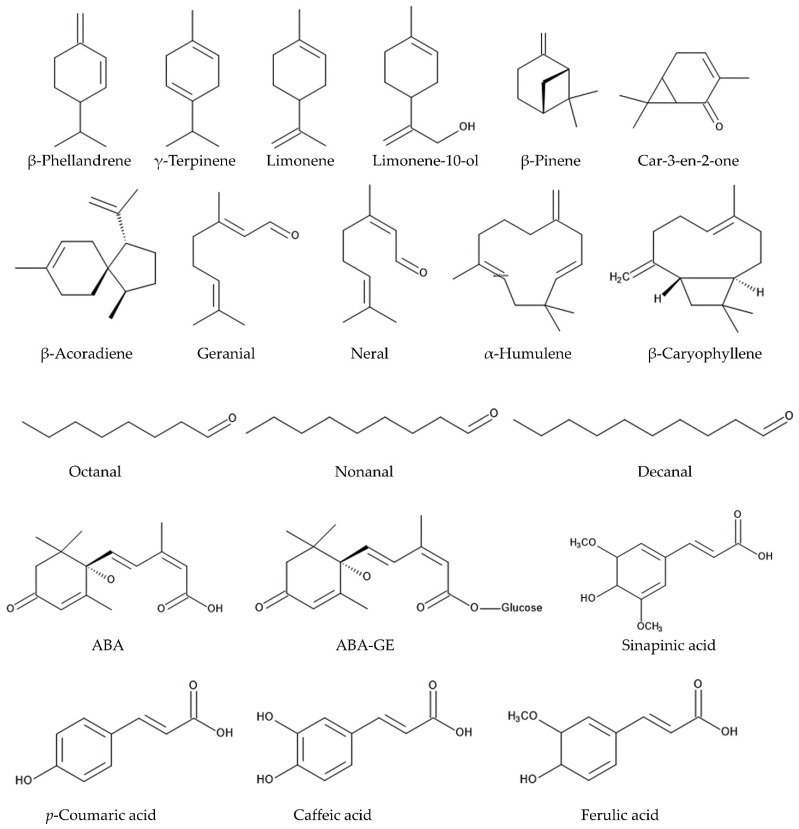
The compounds involved in the allelopathic, nematocidal, insecticidal, and/or anti-fungal activity of citrus fruits.

Vapor from the essential oil of the *Citrus sinensis* fruit peel suppressed the growth of roots and hypocotyls of two weed species, *Euphorbia heterophylla* L. and *Ipomoea grandifolia* (Dammer) O’Donell [76]. The essential oil from the *Citrus sinensis* fruit peel suppressed the germination and seedling growth of *Heliantus annus* L., *Portulaca oleracea* L., *Lupinus albus* L., and *Malva parviflora* L. The major constituent in the essential oil was *d*-limonene [47]. The essential oil of the *Citrus sinensis* fruit peel inhibited the germination and root growth of *Phaseolus lunatus* L. The major constituents in the essential oil were β-acoradiene (12.5%), α-humulene (10.5%), β-pinene (9.6%), car-3-en-2-one (8.4%), and limonene-10-ol (8.3%) [77]. When *Triticum aestivum* (wheat) was grown together with two weed species, *Anagallis arvensis* L. f. *phoenicea* (Scop.) Baumg. and *Malva parviflora,* and the essential oil obtained from the *Citrus sinensis* fruit peel was sprayed on these weeds, the growth of the weeds was suppressed, while the growth and crop yield of *Triticum aestivum* were increased. *d*-Limonene content was 86% of the total essential oil of *Citrus sinensis* fruit peel [78]. These observations suggest that the essential oil of the *Citrus sinensis* fruit peel is allelopathic and contains certain allelochemicals, such as *d*-limonene, β-acoradiene, α-humulene, β-pinene, car-3-en-2-one, and limonene-10-ol. These allelochemicals inhibit the germination and seedling growth of several weed species (Figure 1).

### 2.2. Nematocidal, Insecticidal and Anti-Fungal Activity of Citrus sinensis Fruits

Root-knot nematodes (*Meloidogyne* spp.) make galls in the host plant roots and deprive nutrients from the host plants. *Meloidogyne* spp. are widely distributed in the agricultural field soil, and their host range is wide [79,80,81]. The parasitism of the nematodes causes a significant reduction in agricultural crop production [82,83]. Powder and aqueous extracts of the *Citrus sinensis* fruit peel suppressed the *Meloidogyne incognita* Kofoid & White population and its root gall formation on *Vigna unguiculata* (L.) Walp. under pots and field conditions. β-Caryophyllene and citral (a mixture of geranial and neral) were the major constituents in the powder and extracts [84]. β-caryophyllene was also reported to decrease the infestation of *Meloidogyne incognita* into the roots of *Capsicum annuum* L. and *Solanum lycopersicum* [85,86], causing an increase in the mortality of *Meloidogyne javanica* Treub and *Meloidogyne enterolobii* Yang & Eisenback, and suppressing their egg hatching [87]. The aqueous extracts of the *Citrus sinensis* fruit peel increased the mortality of the second stage of the juveniles of *Meloidogyne incognita* and suppressed the egg hatching and parasitism of *Meloidogyne incognita* into the *Vigna radiata* (L.) R.Wilczek roots [56]. These observations suggest that the powder and aqueous extracts of *Citrus sinensis* fruit peel may contain certain compounds, such as β-caryophyllene and citral, that have nematocidal activity, increase the mortality of nematodes, and suppress their egg hatching and parasitism to the crop plants.

The herbivore insects often cause significant damage to plant growth and vigor, reducing crop production. Thus, an adequate management of the insects is necessary for the agricultural production [88,89,90]. Aqueous extracts of the *Citrus sinensis* fruit peel and seeds increased the mortality of the larvae and adults of the mango mealybug *Drosicha mangiferae* Stebbins [91]. The essential oil obtained from the *Citrus sinensis* fruit peel increased the mortality of the larvae of the tomato borer *Tuta absoluta* Meyrick., while the essential oil did not affect the mortality of the predator of *Tuta absoluta*, *Nesidiocoris tenuis* Reuter [92,93]. Spray application of the essential oil of the *Citrus sinensis* fruits increased the mortality of the larvae and adults of the vine mealybug *Planococcus ficus* Ben-Dov., and limonene was the main constituent of the essential oil [94]. The essential oil of the *Citrus sinensis* fruit peel also showed insecticidal activity against the stored grain pets, such as *Rhyzopertha dominica* Fabricius (lesser grain borer), *Tribolium castaneum* Herbst (red four beetle), *Callosobruchus maculatus* Ficius (cowpea weevil), and *Sitophilus zeamais* Motschulsky (maize weevil) [95,96,97]. Therefore, the aqueous extracts and essential oil of the *Citrus sinensis* fruits may contain certain compounds, such as limonene, that have insecticidal activity and increase the mortality of the herbivore insects.

*Fusarium* species are widely distributed in soil, and some of them cause *Fusarium* diseases, such as necrosis, rot, blight, wilt, and canker on host plant species, which cause a significant reduction in crop production [98,99,100]. Aqueous extracts of the *Citrus sinensis* fruit peel suppressed the growth of the pathogenic *Fusarium oxysporum* (Linford) Snyder & Hansen [101]. The essential oil of the *Citrus sinensis* peel also inhibited the growth of other pathogenic fungi, *Rhizoctonia solani* J.G. Kühn and *Sclerotium rolfsii* Saccardo. Limonene is the main active compound in the essential oil [102]. Therefore, the aqueous extracts and essential oil of *Citrus sinensis* fruit peel may contain certain compounds that have anti-fungal activity and suppress the growth of the pathogenic fungi.

As described in the section, the powder, extracts, and essential oil of the *Citrus sinensis* fruits possess nematocidal, insecticidal, and/or anti-fungal activity and may contain certain compounds involved in these activities.

## 3. *Citrus reticulata* (Mandarin)

*Citrus reticulata,* containing mandarin and tangerine, is thought to be one of the basic taxa of *Citrus* species and to be distributed from China and Southeast Asia [2,4,6,103]. The fruits of the *Citrus reticulata* are mostly consumed fresh, but some of the fruits are processed into juices and other foods. The processing fruit waste was about 64% of the total fresh fruit weight, including peel (76% of total dry fruit weight), pulp (23%), and seeds (0–1%) [9].

Aqueous and methanol extracts of the *Citrus reticulata* fruits inhibited the germination and seedling growth of *Lactuca sativa*, and the inhibitory activity of the methanol extracts was greater than that of the aqueous extracts [104]. Aqueous methanol extracts of the *Citrus reticulata* fruit peel inhibited the germination of *Echinochloa crus-galli,* and a considerable amount of abscisic acid was found in the extracts [105]. Abscisic acid is one of the plant hormones and has germination and growth inhibitory activity [106,107]. The essential oil obtained from the flavedo (part of peel) of the *Citrus reticulata* fruits inhibited the germination and growth of *Raphanus sativus* L. and the spore germination and cell division of the moss *Tortula muralis* Hedw. The main active constituent in the essential oil was limonene [103]. Essential oil of the *Citrus reticulata* fruit peel also suppressed the germination and seedling growth of *Heliantus annus*, *Portulaca oleracea*, *Lupinus albus*, and *Malva parviflora*. The major constituents in the essential oil were *d*-limonene and γ-terpinene [47].

The essential oil of the *Citrus reticulata* fruit peel inhibited egg hatching and increased the motility of larvae of the root-knot nematode *Meloidogyne incognita*. The main active constituent in the essential oil was limonene [108]. The essential oil of the *Citrus reticulata* fruit peel increased the mortality of the larvae of the tomato borer *Tuta absoluta,* while the essential oil did not affect the mortality of its predator, *Nesidiocoris tenuis.* The main constituent in the essential oil was limonene [92,93]. The essential oil of the *Citrus reticulata* fruit peel increased the mortality of the stored grain pets, *Sitophilus zeamais* (maize weevil) and *Trogoderma granarium* Everts (cabinet beetle) [96,109]. Limonene was the main active constituent in the essential oil [109].

The essential oil of the *Citrus reticulata* fruit peel inhibited the growth and spore formation of five plant pathogenic fungi, *Alternaria alternata* (Fr.) Keissl., *Rhizoctonia solani*, *Curvularia lunata* R.R. Nelson & Haasis, and *Helminthosporium oryzae* S.Ito & Kurib. Drechsler ex Dastur, including *Fusarium oxysporum,* and the major constituents in the essential oil were limonene (46.7%), geranial (19.0%), and neral (14.5%) [110]. The essential oil obtained from the flavedo of the *Citrus reticulata* fruits inhibited the growth of *Penicillium italicum* Wehmer and *Penicillium digitatum* (Pers.) Sacc. [111], which cause post-harvest disease in the stored fruits [112]. The major active constituents in the essential oil were octanal, nonanal, decanal, and limonene [111].

These observations suggest that the extracts and essential oil of the *Citrus reticulata* fruits may contain certain compounds, such as abscisic acid, limonene, γ-terpinene, geranial, neral, octanal, nonanal, and decanal, which have allelopathic, nematocidal, insecticidal, and/or anti-fungal activity (Figure 1).

## 4. *Citrus limon* (Lemon)

*Citrus limon* is thought to be a multi-hybrid of *Citrus reticulata, Citrus maxima,* and *Citrus medica* and is distributed from Southeast Asia [2,4,6,103]. The processing fruit waste was about 62% of the total fresh fruit weight, including peel (70% of total dry fruit weight), pulp (25%), and seeds (5%) [9].

The aqueous ethanol extracts of the *Citrus limon* fruit peel suppressed the growth and cell division of *Allium cepa* roots [66]. The aqueous extracts of the *Citrus limon* fruit peel increased the mortality of the second stage of juveniles of *Meloidogyne incognita* and suppressed the egg hatching and parasitism of *Meloidogyne incognita* into the *Vigna radiata* roots [56]. The essential oil of the *Citrus limon* fruit peel increased the mortality of the larvae of the tomato borer *Tuta absoluta,* while the essential oil did not affect the mortality of its predator, *Nesidiocoris tenuis.* The major constituent in the essential oil was limonene [92,93]. Spray application of the essential oil of the *Citrus limon* fruit peel increased the mortality of the larvae and adults of the vine mealybug *Planococcus ficus,* and limonene was the main constituent of the essential oil [94]. The essential oil of the *Citrus limon* fruit peel increased the mortality of the stored grain pets, *Rhyzopertha dominica* and *Sitophilus granaries* Linnaeus (grain weevil) [102,113]. The essential oil of the *Citrus limon* fruit peel inhibited the growth of pathogenic fungi, *Rhizoctonia solani* J.G. Kühn and *Sclerotium rolfsii.* Limonene is the main active compound in the essential oil [102]. These observations suggest that the extracts and essential oil of the *Citrus limon* fruit peel may contain certain compounds, including limonene, which have allelopathic, nematocidal, insecticidal, and/or anti-fungal activity.

## 5. *Citrus aurantiifolia* (Lime)

*Citrus aurantiifolia* is thought to be a hybrid between *Citrus medica* L. and *Citrus micrantha* Wester and is distributed from tropical Southeast Asia [2,4,6,12]. The processing fruit waste was 11–12% of the total fresh fruit weight for peel and 40–45% of the total fresh fruit weight for pulp [11]. Aqueous and methanol extracts of the *Citrus aurantiifolia* fruits inhibited the germination and seedling growth of *Lactuca sativa*, and the inhibitory activity of the methanol extracts was greater than that of the aqueous extracts [104]. The essential oil of the *Citrus aurantiifolia* fruit peel increased the mortality of the stored grain pets, *Sitophilus zeamais* (maize weevil), and limonene was the main active constituent in the essential oil [109]. These observations suggest that the extracts and essential oil of the *Citrus limon* fruits may contain certain compounds, including limonene, which have allelopathic and/or insecticidal activity.

## *6. Citrus maxima* (Pomelo)

*Citrus maxima,* bearing the largest fruits among *Citrus* species, is thought to be one of the basic taxa of *Citrus* species and is distributed from Southeast Asia [2,4,6]. The processing fruit waste was 36–40%, 26–32%, and 3–4% of the total fresh fruit weight for the peel, pulp, and seeds, respectively [11]. Aqueous and methanol extracts of the *Citrus maxima* fruits inhibited the germination and seedling growth of *Lactuca sativa*, and the inhibitory activity of the methanol extracts was greater than that of the aqueous extracts [104]. The observation suggests that the extracts of the *Citrus maxima* fruits may contain certain compounds that have allelopathic activity.

## 7. *Citrus paradisi* (Grapefruit)

*Citrus paradisi* is thought to be a natural hybrid between *Citrus maxima* and *Citrus sinensis* and was discovered in the Caribbean [2,4,6]. The processing waste is about 62% of the total fresh fruit weight, including the peel (65% of total dry fruit weight), pulp (33%), and seeds (2%) [9]. The aqueous extracts of the *Citrus paradisi* fruit peel increased the mortality of the second stage of the juveniles of *Meloidogyne incognita* and suppressed their egg hatching and parasitism into the *Vigna radiata* roots [56]. The essential oil of the *Citrus paradisi* fruit peel also inhibited the growth of the pathogenic fungi *Rhizoctonia solani* J.G. Kühn and *Sclerotium rolfsii* (Sacc.) Redhead & S-T. Mullineux. Limonene is the main active compound in the essential oil [102]. These observations suggest that the extracts and essential oil of *Citrus paradisi* peel may contain certain compounds that have allelopathic, insecticidal, and/or anti-fungal activity.

## 8. *Citrus aurantium* (Sour Orange)

*Citrus aurantium* is thought to be a natural hybrid between *Citrus maxima* and *Citrus reticulata* and is distributed from Southeast Asia [2,4,6,114]. The *Citrus aurantium* fruits are processed into juices, functional foods, and food additives [115,116].

Four weed species, *Cynodon dactylon* L., *Chenopodium album, Avena sativa* L., and *Amaranthus retroflexus* L., which naturally occur in the fields researched, did not grow well under the trees of *Citrus aurantium.* Light, water, nutrients, and soil pH conditions under the trees did not disturb the emergence and growth of these weed species. However, the soil under the *Citrus aurantium* trees, the aqueous extracts of the decaying materials of the trees, and the volatiles from the trees suppressed the emergence and seedling growth of these weed species [117]. The observation suggests that the soil may contain certain allelochemicals, which may be leased during the decaying process of the plant materials, including its fruits. Some other allelochemicals may also be released from the trees as volatiles. The essential oil of the *Citrus aurantium* fruit peel also suppressed the germination and seedling growth of *Heliantus annus*, *Portulaca oleracea*, *Lupinus albus*, and *Malva parviflora*. The major constituent in the essential oil was *d*-limonene [47].

Methanol extracts of the *Citrus aurantium* fruit peel and seeds suppressed the egg hatching and increased the mortality of the parasitic *Meloidogyne incognita* (root-knot nematode), *Rotylenchulus reniformis* Linford and Oliveira (reniform nematode), and *Tylenchulus semipenetrans* Cobb. (citrus nematode) [118]. Petroleum ether extracts of the *Citrus aurantium* fruit peel increased the mortality of the adults of the olive fruit fly *Bactrocera oleae* Rossi and the Mediterranean fruit fly *Ceratitis capitata* Wiedemann [119,120]. Aqueous extracts of the *Citrus aurantium* fruit peel and seeds increased the mortality of the larvae and adults of the mango mealybug *Drosicha mangiferae* [91]. The essential oil of the *Citrus aurantium* fruit peel increased the mortality of four aphid species, such as *Acyrthosiphon pisum* Harris (pea aphid), *Rhopalosiphum padi* Linnaeus (bird cherry coat aphid), *Aphis fabae* Scopoli (black bean aphid), and *Macrosiphum euphorbiae* Thomas (potato aphid). When the essential oil was applied to the *Aphis fabae*-infested *Phaseolus* plants in the sealed container, 72% of the aphids were dead. Limonene was the main constituent of the essential oil [121]. The essential oil of the *Citrus aurantium* fruit peel increased the mortality against stored grain pets, such as *Sitophilus granaries* Linnaeus (wheat weevil), *Tribolium castaneum* (red four beetle), *Cryptolestes ferrugineus* Stephens (flat bark beetle), and *Liposcelis bostrychophila* Badonnel (booklouse), and showed the anti-fungal activity against pathogen fungus *Fusarium oxysporum*. Limonene was the major constituent of the essential oil, taking 74–97% of the essential oil of the *Citrus aurantium* fruits obtained from several different regions and countries. Limonene also increased the mortality of these sored grain pets at concentrations greater than 25 μL per L of air volume [122]. These observations suggest that the extracts and essential oil of *Citrus aurantium* fruits and soil under the *Citrus aurantium* tree may contain certain compounds that have allelopathic, insecticidal, and/or anti-fungal activity.

## 9. *Citrus margarita* (Kumquat, Syn. *Fortunella margarita*)

*Citrus margarita* is thought to be one of the true *Citrus* species and is distributed from Southeast China [123]. The essential oil of the fruit peel suppressed the root growth of *Helianthus annuus,* and limonene (22%), β-phellandrene (12%), and β-pinene (11%) were major constituents of the essential oil [124] (Figure 1).

## 10. *Citrus junos* (Yuzu)

*Citrus junos* is thought to be a hybrid between *Citrus ichangensis* Y.Ye, X.Liu, S.Q.Ding & M.Liang and *Citrus reticulata* var. *austere*, and it is distributed from China and cultivated in East Asia, South Europe, and Australia [125,126]. The fruit is processed into juices as an ingredient in sauces for its special flavor and other functional foods [127,128].

The growth of two weed species, *Sonchus oleraceus* and *Digitaria ciliaris* (Retz.) Koel, was suppressed when these species were grown in the soil mixed with the peel powder of the *Citrus junos* fruits [129]. The powder of the *Citrus junos* fruit waste obtained from the food processing industry also inhibited the growth of three weed species: *Digitaria sanguinalis*, *Phleum pratense* L., and *Lolium multiflorum* Lam [45]. Ethanol extracts of the *Citrus junos* fruit peel inhibited the growth of *Chenopodium quinoa* Willd. [129]. Aqueous methanol extracts of the peel, pulp, and seeds of the *Citrus junos* fruit waste also inhibited the growth of *Digitaria sanguinalis*, *Phleum pretense,* and *Lolium multiflorum*. The inhibitory activity of the peel extracts was greatest, followed by that of the pulp extracts and seed extracts [130]. These observations suggest that the *Citrus junos* fruit waste is allelopathic, and the powder and extracts of the fruit waste may contain certain allelochemicals. The allelopathic activity of the peel may be greater than that of pulp and seeds.

The aqueous methanol extracts of the *Citrus junos* fruit peel were separated through the bioassay-guided purification process, in which the allelopathic activity of all fractions obtained after each separation step was evaluated, and the most active fraction was subjected to the next separation step. The purification process made the isolation of a most active allelopathic substance, and the isolated compound was identified as abscisic acid-β-D-glucopyranosyl ester (ABA-GE) [131]. ABA-GE concentration was 23.5 μg/g, 7.2 μg/g, and 1.1 μg/g dry weight of the fruit processing waste for the peel, pulp, and seeds, respectively, and these concentrations were consistent with the inhibitory activity of these extracts [130]. In addition, ABA-GE was found in other citrus fruit peel, such as *Citrus unshiu* (Swingle) Marcow., *Citrus sudachi* Hort. ex Shirai, and *Citrus hassaku* Group, and was responsible for the allelopathic activity of these fruit peel [132,133].

Isolated ABA-GE inhibited the growth of *Digitaria sanguinalis*, *Phleum pretense,* and *Lolium multiflorum* seedlings at concentrations greater than 0.1 μM. The growth inhibitory activity of ABA-GE on these weed species was 26–40% of that of abscisic acid (ABA) [134]. ABA-GE was thought to be a physiologically inactive conjugated ABA form [135,136]. However, an apoplastic ABA-β-D-glucosidase, which releases free ABA from ABA-GE, has been found in several plant species, including *Digitaria sanguinalis*, *Phleum pretense, Lolium multiflorum,* and *Arabidopsis thaliana* L. [132,134,137,138,139]. As the isolated ABA-GE showed growth inhibitory activity on these weed species, it is possible that ABA is released from ABA-GE by their ABA-β-D-glucosidase, and the liberated ABA causes growth inhibition.

These observations suggest that the *Citrus junos* fruits and fruit waste are allelopathic and contain certain allelochemicals. The most active allelochemical was isolated and identified as ABA-GE. ABA-GE showed growth inhibitory activity against several weed species. The fruits of other *Citrus* species, such as *Citrus unshiu, Citrus sudachi,* and *Citrus hassaku* are also allelopathic and contain ABA-GE as an allelochemical [132,133] (Figure 1).

## 11. Sustainable Management of Citrus Fruit Waste and Active Compounds in the Waste

Based on the literature review, this work identified that all *Citrus* species described in this paper showed that their fruits and peel powder, extracts, and essential oil have allelopathic (or herbicidal), nematocidal, insecticidal, and/or anti-fungal activity and may contain certain compounds involved in these activities. The activities of the extracts and the essential oil of the fruit peel were most reported, and limonene was the most frequently identified compound in the extracts and the essential oil of the peel of the citrus fruits.

**Figure 2:**
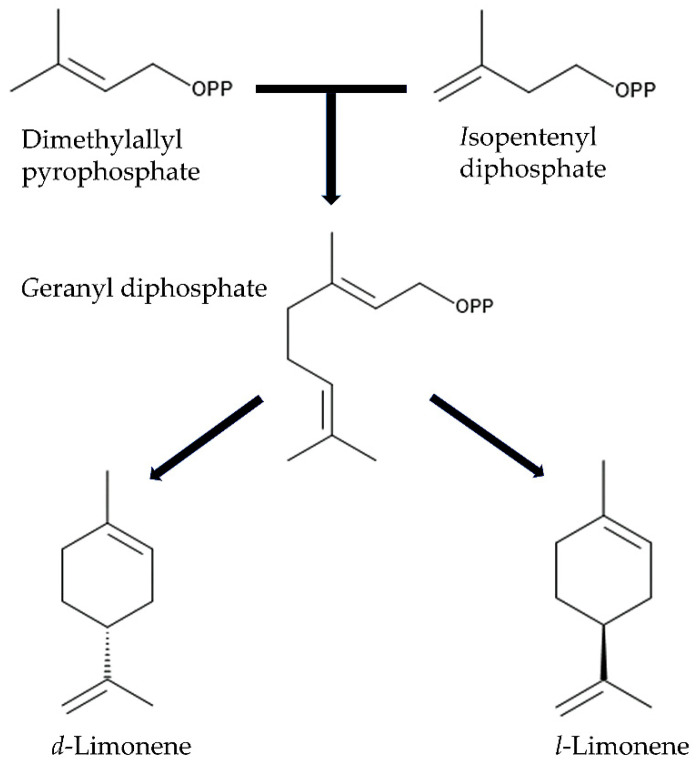
Biosynthetic pathway of *d*-limonene and *l*-limonene.

Limonene is the main constituent in the essential oil, ranging from 49% to 90% of the total essential oil of *Citrus sinensis*, *Citrus reticulata*, *Citrus limon,* and *Citrus aurantium* [140,141]. Limonene is a cyclic monoterpene synthesized from geranyl diphosphate and exists in nature as two enantiomers, *d*-limonene [(*R*)-(+)-limonene] and *l*-limonene [(*S*)-(−)-limonene], which are catalyzed by *d*-limonene synthase and *l*-limonene synthase, respectively [142,143,144]. *d*-Limonene and *l*-limonene have different odors, and citrus fruits primarily contain *d*-limonene and little or no *l*-limonene [145,146,147]. *d*-Limonene, with about 100 minor components, contributes to the respective flavor of different citrus fruits [146]. *d*-Limonene was reported to show several pharmacological effects, such as anti-cancer activity, neuroprotective activity, hypoglycemic activity, analgesic activity, anti-inflammatory activity, antimicrobial activity, and hygiene control, and *d*-limonene has been classified as a low toxic compound against animal cells [148,149]. Limonene, likely *d*-limonene, in the extracts and the essential oil of the citrus fruit peel also showed allelopathic, nematocidal, insecticidal, and anti-fungal activity, as described in the above section [47,67,92,102,103] (Figure 2).

β-Caryophyllene was found in the extracts of the *Citrus sinensis* fruits as a nematocidal active substance [84] and was also reported to possess allelopathic activity against several plant species due to causing oxidative stress conditions [150,151]. ABA-GE was identified in the *Citrus junos* fruits and fruit processing waste and showed allelopathic activity against several weed species [129,130,131,132,133,134]. The accumulation of ABA-GE occurs in certain plant tissues, including plant fruits, in response to various environmental conditions and fruit maturation [135,136]. The increase in the ABA-GE concentration in the citrus fruit peel was observed as fruit maturation [133]. A considerable amount of ABA-GE accumulation of *Citrus sinensis* in the exocarp (outer part of peel) was also observed [152,153]. Ferulic acid, caffeic acid, *p*-coumaric acid, and sinapinic acid were identified in several citrus fruits [154,155]. These compounds were also identified in many other plant species and reported to act as allelochemicals, causing germination and growth inhibition [156,157,158]. Several other compounds were found in the extracts and the essential oil of the citrus fruits. These compounds, including *d*-limonene, β-caryophyllene, ABA-GE, ferulic acid, caffeic acid, *p*-coumaric acid, and sinapinic acid, may be involved in the allelopathic, nematocidal, insecticidal, and/or anti-fungal activity of the extracts and the essential oil of the citrus fruits. The existence of some of these compounds, such as limonene, β-pinene, γ-terpinene, geranial (*E*-citral), decanal, ferulic acid, caffeic acid, *p*-coumaric acid, sinapinic acid, and ABA-GE was also confirmed in the citrus fruit processing waste [21,130].

The weeds, nematodes, herbivore insects, and pathogenic fungi cause significant crop yield loss, and adequate management is necessary to achieve the potential yield in agricultural production [60,64,81,90,99]. The extracts and essential oil of citrus fruits, including the fruit waste, inhibited the germination and growth of several weed species; suppressed the growth of nematodes, herbivore insects, and pathogenic fungi; and increased the mortality of nematodes and herbivore insects. The citrus fruit peel incorporated into the soil showed allelopathic and nematocidal activity, and a spray containing the extracts and essential oil of the citrus fruits also showed allelopathic and insecticidal activity. The citrus fruit processing waste was also confirmed to contain the active compounds for these activities. Therefore, the extracts and essential oil of the citrus fruit waste and the waste itself are potentially useful for the management of weeds, nematodes, herbivore insects, and pathogenic fungi in some agricultural settings, such as mulch, soil additive, and folier spray to reduce commercial herbicide dependency.

## 12. Conclusions

Citrus fruit processing produces a large amount of waste, causing environmental issues. The development of herbicidal, nematocidal, insecticidal, and anti-fungal materials using citrus fruit processing waste may be one of the solutions to minimize the processing waste and reduce the environmental impacts. The citrus fruit waste contains several functional compounds, but the processing requires adequate production facilities. The transportation of citrus waste is also not cost-effective for distant sites. The main target of the waste, such as herbicidal, nematocidal, insecticidal, and anti-fungal materials, is for agricultural production in the proximity of the citrus fruit processing factories. Large-scale field observations are necessary in the future to evaluate the effectiveness of citrus fruit processing waste as herbicidal, nematocidal, insecticidal, and anti-fungal materials.

## Data Availability

No new data were created or analyzed in this study.
